# Challenges in the Prevention and Management of Diabetic Kidney Diseases

**DOI:** 10.3389/fcdhc.2021.728320

**Published:** 2021-08-23

**Authors:** Alper Sonmez

**Affiliations:** Department of Endocrinology and Metabolism, Gulhane Faculty of Medicine, University of Health Sciences, Ankara, Turkey

**Keywords:** diabetic nephropathy, diabetic kidney disease, diabetes mellitus, clinical inertia, cardiovascular risk

*“If you define the problem correctly, you almost have the solution”*

Steve Jobs

## Background

Diabetic kidney disease (DKD) is one of the common complications of diabetes mellitus, which substantially decreases the quality of life and increases the risk of premature mortality (1). Although it is the most common cause of end-stage renal disease (ESRD) (2), the mortality is mostly due to cardiovascular diseases and therefore DKD is regarded as a major cardiovascular risk factor (3, 4). Due to its chronic and slowly progressing nature, DKD is generally diagnosed by screening tests showing albuminuria or low eGFR, or both in subjects with diabetes. Up to one-third of patients with type 1 diabetes (T1DM) (5–7)and nearly half of patients with type 2 diabetes (T2DM) have DKD (6, 8–10). Yet, fewer of them receive optimal care to prevent DKD progression and avoid the cardiovascular and renal endpoints (11).

Although the term “Diabetic Nephropathy” is used interchangeably with DKD, the former more specifically describes the histological alterations such as glomerular basement membrane thickening, or mesangial proliferation observed in subjects with T1DM, which predominantly occurs due to chronic hyperglycemia (12). However, DKD observed in subjects with T2DM involves Diabetic Nephropathy but also the alterations seen due to other causes such as aging, hypertension, or obesity. This is probably the reason for observing DKD more frequently in subjects with T2DM. As multiple risk factors play role in the pathogenesis of DKD and its cardiovascular consequences, intensive glucose control is not enough to prevent renal and cardiovascular endpoints in DKD (13). Therefore, chronic risk management is essential along with good glycemic regulation, to prevent the occurrence and progression of DKD and to reduce the premature cardiovascular events in patients with diabetes (14–17).

Unfortunately, the global data shows that people with diabetes are not under good glycemic control, nor they attain the metabolic targets (18–20). We have recently performed a nationwide survey in Turkey (6). Our findings replicate the global data and show that less than half of patients with T2DM reach the target HBA1c levels and only 10% of them simultaneously attain the targets for blood glucose, blood pressure, LDL Cholesterol. The situation is even worse in patients with T1DM, with less than 5% attainment rates of the three targets. When we also consider smoking cessation and regular exercise, only 1.5% of patients with both types of diabetes reach all these targets simultaneously (6). There is also significant inertia in establishing appropriate targets and optimizing treatment to achieve treatment goals (11, 21, 22). To solve this problem, we should better understand the reasons behind it.

## Diabetic Kidney Disease (DKD): Scope of the Problem

There are significant problems in the current practice of diabetes management involving the screening of DKD, optimization of therapy, making timely referrals, and managing risk factors and complications (23). The failure to establish appropriate targets and modify treatment to attain the goals, namely “*clinical inertia*” is responsible for the increased complications and health care burden (11, 24). Some of the main barriers to optimal patient care are touched upon below:

### Patient-Related Factors

The capacity of patients to obtain, process, and understand basic health information, so-called “health literacy”, is one of the key factors in the appropriate management of patients with diabetes (25, 26). With diabetes in general have unhealthy lifestyles, which are not easy to modify (27). Older adults and patients with lower socio-economic status are more likely to have diabetes (28, 29) and less likely to follow the recommendations of their physicians (30). Many people with diabetes are not intended to use insulin due to the fear of hypoglycemia or weight gain or becoming dependent on insulin treatment (31, 32). Negative media coverage is also a significant reason for the incompliance especially of statins (22, 33, 34). Polypharmacy is also a major obstacle in reducing patient compliance and the attainment of targets (35).

### Physician-Related Factors

Diabetes is so common that most of the patients are inevitably followed up in primary or secondary care facilities. Physicians working in these services may not have enough knowledge or experience in setting appropriate targets or implementing medications or modifying treatment while managing patients with diabetes (11). Low GFR is often not considered for adjusting the dose of antidiabetic drugs that are contraindicated in DKD (36). Also, physicians may not care enough to modify the doses of antidiabetic and antihypertensive medications during the follow-up of patients with diabetes (21). Lack of enough time to communicate with the patients and lack of supporting health staff are always the leading barriers in the crowded outpatient units (23). There are a multitude of diabetes guidelines to fill the knowledge gap in the field (14–17). However, physicians working in crowded outpatient units are bewildered by these long, complicated, and hard-to-read documents. They need concise, and problem-oriented algorithms to help them find their way.

### Healthcare System

High quality and sustainable health care depend on well-adjusted infrastructures of the healthcare system, software for the electronic data recording and follow-up, wide-ranging health insurance coverage, and a reasonable reimbursement strategy (37). There are huge differences between the costs of new and old antihyperglycemic medications, which is not easy to be afforded by the patients (38). All these factors significantly differ between different countries. Doctors lack enough time to spend with their patients. The centers may not have enough capacity for necessary laboratory tests and many physicians do not have a consultation link for further management of the complications of their patients.

## Grand Challenge in the Prevention and Management of DKD and Its Consequences

We need to cross all these barriers to optimize the management of patients with DKD and prevent its severe outcomes. Strategies should be implemented at every level for judicious patient care. Below I’d like to mention these strategies in brief.

### Patient-Level Strategies

The higher the education of the patients, the better the attainment of the treatment targets (6). Therefore, every effort should be spent to increase public health literacy. Moreover, patients with diabetes and their families should be informed about the possible consequences of diabetes, the side effects of medications, and the ways of handling acute and chronic complications. More attention should be paid to the vulnerable patients for the risk of DKD and its consequences, such as the older adults and the patients with lower socioeconomic status. As the establishment of a healthy lifestyle is the key element of better patient care, all patients should be encouraged for healthy eating, performing exercise, and smoke cessation. Also, patients should be assisted by judicious websites and social media pages, which are designed to give evidence-based, comprehensible, and non-biased information about diabetes and its complications. The media should be more sensitive to cover the news about medical and pharmacological innovations and improvements.

### Physician-Level Strategies

The prevalence of diabetes is substantially increased in recent decades. Therefore, the graduates of the medical schools should be well educated about the diagnosis and management of diabetes mellitus. Also, postgraduate courses or certification programs should be implemented regularly to improve the information and experience of the physicians working in the field. Lengthy guidelines should be summarized to the rule of thumb brochures for the busy physicians to guide them for the screening, diagnosis, and management of patients with diabetes.

### Strategies to Improve Healthcare

To increase the time spent for each patient and to better educate them, interconnected healthcare teams equipped with skilled physicians, nurses, dietitians, podiatrists, and social workers should be established. Intelligent electronic health registries should be implemented not only to store data but also to alert the physicians when to screen for DKD, how to apply appropriate medications, and when to refer patients to nephrologists. The health insurance coverage should be disseminated and the drugs with cardioprotective functions should be reimbursed in general. To improve public healthcare strategies, real-world evidence about health economics should be processed and published. Also, clinical studies should be undertaken to develop biomarkers for the early detection of patients with a high risk of developing DKD.

## Objectives of the Diabetic Nephropathy Special Issue

**Figure 1:**
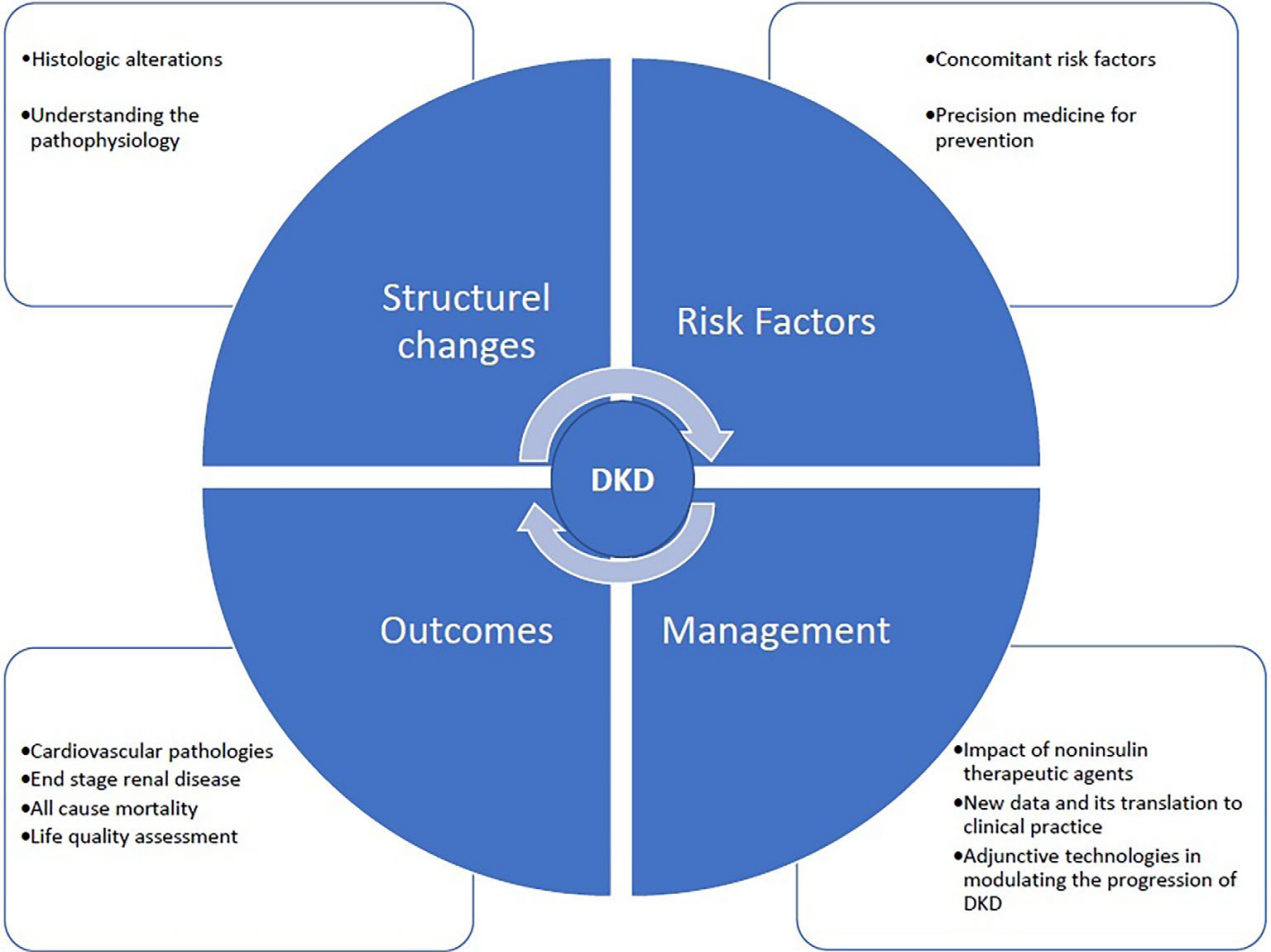
Potential fields of publication which will be covered by Diabetes Nephropathy.

The critical mission of Frontiers in Clinical Diabetes and Healthcare section on Diabetes Nephropathy is to provide a global network between the basic and clinical scientists and the clinicians. The fast and easy publication platform of the journal helps the authors to quickly submit their work, which will immediately be evaluated by the distinguished editors and referred to the expert referees without wasting time. Our dedication is to unite the basic and clinical information and radiate them to the physicians working in the field to help improving the management of diabetes and preventing its ominous complication, DKD. The potential fields of publication that will be covered by *Diabetes Nephropathy* are given in **Figure 1**.

## Author Contributions

The author confirms being the sole contributor to this work and has approved it for publication.

## Conflict of Interest

The author declares that the research was conducted in the absence of any commercial or financial relationships that could be construed as a potential conflict of interest.

## Publisher’s Note

All claims expressed in this article are solely those of the authors and do not necessarily represent those of their affiliated organizations, or those of the publisher, the editors and the reviewers. Any product that may be evaluated in this article, or claim that may be made by its manufacturer, is not guaranteed or endorsed by the publisher.
